# Cooperation between cancer cells

**DOI:** 10.1093/emph/eoy003

**Published:** 2018-01-23

**Authors:** Marco Archetti

**Affiliations:** School of Biological Sciences, University of East Anglia, Norwich, UK

## INTRA-TUMOR COOPERATION

**Figure 1.**
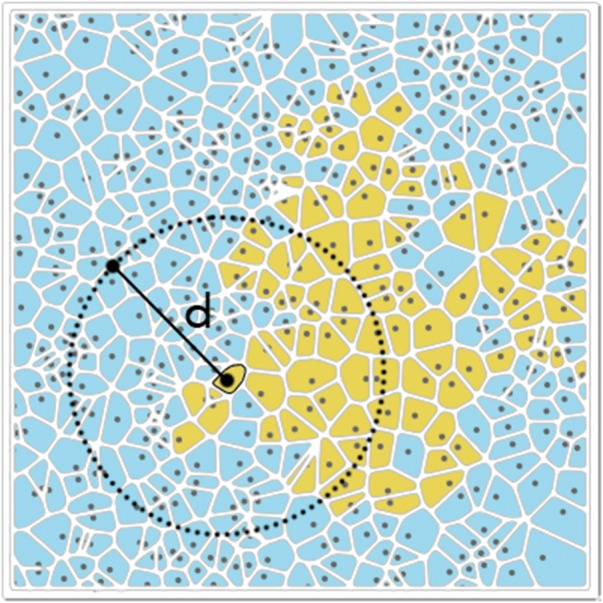
A monolayer of producer (blue) and non-producer (yellow) cells of a growth factor with diffusion range *d*

Cancer cells secrete growth factors that induce proliferation, protect against apoptosis and the immune system or promote neo-angiogenesis [1]. As they are diffusible (Fig. 1), the growth factors produced by a cell can be used by other neighbouring cells, an example of cooperation among cancer cells [2].

A conceptual problem arises: a non-producer cell can exploit the growth factors secreted by its neighbouring producer cells without paying the cost of production; hence non-producers should have a proliferation advantage and spread in the population. How can intra-tumour cooperation be maintained then? Why do not non-producer mutants drive producer cells to extinction?

## EVOLUTIONARY PERSPECTIVES

**Figure 2.**
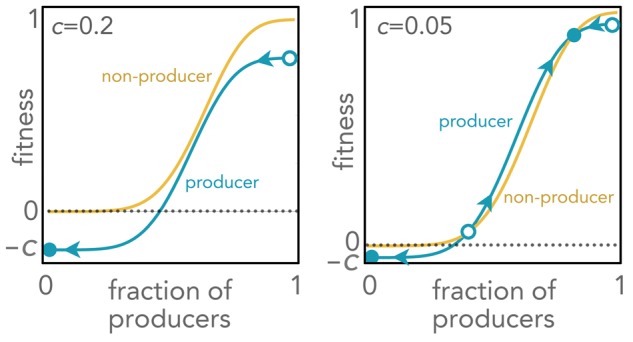
Fitness of producer and non-producer cells as a function of the fraction of producer cells for different costs of growth factor production *c*. Equilibria (full circles: stable; open circles: unstable) and the direction of the dynamics (arrows) are shown

In some cases, two clones producing one growth factor each can coexist if both are essential, because the two clones depend on each other [3], similar to mutualism between species. More in general, in cases without such mutual dependence, a clone producing a growth factor can coexist with a clone producing the same growth factor at a lower (or null) rate if its effect is a sigmoid function of its concentration (which is common for growth factors) [4]: in this case, producer cells have a proliferation advantage at intermediate frequencies—leading to a stable mixed equilibrium of the two types (Fig. 2, right panel). Cooperation collapses if the cost/benefit of the growth factor is high enough (Fig. 2, left panel). The dynamics of growth factor production can be studied using evolutionary game theory and experimental evolution [4].

## FUTURE IMPLICATIONS

Targeted therapies aiming at impairing cooperation by blocking growth factors or their receptors [5] are prone to the evolution of resistance. Understanding intra-tumour cooperation is essential to develop evolutionarily stable therapies. An alternative approach could be to use autologous cancer cells in which genes for growth factors have been knocked out [6].
